# Accuracy and Efficiency of Fusion Robotics™ Versus Mazor-X™ in Single-Level Lumbar Pedicle Screw Placement

**DOI:** 10.7759/cureus.15939

**Published:** 2021-06-26

**Authors:** Mohamed A Soliman, Asham Khan, Timothy E O'Connor, Kevin Foley, John Pollina

**Affiliations:** 1 Department of Neurosurgery, Jacobs School of Medicine and Biomedical Sciences at University at Buffalo, Buffalo, USA; 2 Department of Neurosurgery, Buffalo General Medical Center, Kaleida Health, Buffalo, USA; 3 Department of Neurosurgery, Faculty of Medicine, Cairo University, Cairo, EGY; 4 Department of Neurosurgery, Semmes-Murphey Clinic & University of Tennessee Health Science Center, Memphis, USA

**Keywords:** robot-assisted spine, pedicle screws, surgical workflow, screw accuracy, mazor-x, fusion robotics

## Abstract

Introduction

There has been a surge in robot utilization in spine surgery over the past five years with the rapid development of new spine robotic platforms. This study aimed to compare a new robotic spine platform from Fusion Robotics^TM ^(Fusion Robotics, Helena, MT) with the widely used Mazor-X^TM^ Stealth Edition robotic platform (Medtronic, Dublin, Ireland) in terms of workflow and lumbar pedicle screw placement accuracy.

Methods

A cadaver lab was conducted, which included four procedures with single-level lumbar pedicle screw placement using the Fusion Robotics^TM^ system. These four procedures were compared to four propensity-score matched cases with single-level lumbar pedicle screw placement using the Mazor-X^TM^ Stealth Edition. A single surgeon performed all surgeries. The cases were matched in terms of demographics (age, sex, race, BMI) and comorbidities (Charlson Comorbidity Index score). The primary outcome measure was the operative workflow efficiency (duration as measured with a stopwatch by an independent observer). The secondary outcome measures were pedicle screw accuracy and accuracy to plan.

Results

After propensity-score matching, there were four cases in each group with no significant between-group differences in terms of sex, race, BMI, or surgical levels; however, there were significant differences in terms of age (p=0.01) and comorbidities (p<0.001). The workflow efficiency measurement showed that the Fusion Robotics^TM^ platform had a significantly shorter duration in terms of the system set-up time, planning to in-position time, and total procedure time (p<0.05). However, there was no significant difference between the robotic platforms in terms of creating a sterile barrier, scanning and importing images, creating a plan, screw placement, screw accuracy, and screw accuracy to plan.

Conclusion

Based on our findings, the Fusion Robotics^TM^ platform had a significantly shorter procedure workflow duration while maintaining the same accuracy as the most commonly used robotic platform (Mazor-X^TM^). This is the first study to directly compare different spine surgery robotic systems.

## Introduction

There has been a surge in robot utilization in surgeries in the last 20 years in many surgical disciplines such as general surgery, urology, and gynecology [1]. In 2004, the SpineAssist® robot (Mazor Robotics Ltd., Caesarea, Israel) became the first such system to be employed in spine procedures after gaining FDA approval [2]. However, many technical problems were encountered with the original device, such as altered accuracy of the robot arm, poor registration between the pre and intraoperative images, and problems related to clamp attachment to the spinous process [3,4]. However, these technical difficulties were addressed in the new Mazor-X^TM^ Stealth Edition (Medtronic, Dublin, Ireland) [5]. Currently, three robotic systems are available to be used in spine surgery: ExcelsiusGPS® (Globus Medical, Inc., Audubon, PA), ROSA ONE® (Zimmer Biomet, Brognard, France), and the Mazor-X^TM^ Stealth Edition [6]. The Mazor-X^TM^ Stealth Edition is the most widely used system and the one that is predominantly featured in the literature, as Mazor was the first to develop a robotic spine platform [6]. All of the above platforms use the shared-control robotic system, and none of them uses the supervisory-controlled interaction or telesurgical interaction systems [7]. According to GlobalData, the number of robotic procedures is expected to increase annually by 6.5-12% from 2020 to 2030 [8]. Given this projected growth, it is not surprising that other companies are developing robotic spine systems as well. In this study, we compared a new robotic spine platform from Fusion Robotics^TM ^(Fusion Robotics, Helena, MT) to the widely used Mazor-X^TM^ robotic platform in terms of workflow and screw placement accuracy.

## Materials and methods

We have adhered to the Strengthening The Reporting of OBservational studies in Epidemiology (STROBE) guidelines in conducting this study and reporting our findings [9].

Demographics

Fusion Robotics^TM^ Group

We conducted a lab study using two cadavers, and each specimen underwent two separate procedures (L2-3 and L4-5 pedicle screw placement). The cadavers’ demographics (age, sex, race, BMI) and comorbidities (Charlson Comorbidity Index score) were collected from the donor summary report.

Mazor-X^TM^ Stealth Edition Group

The electronic health records of patients who had undergone a single-level robotic-assisted lumbar fusion between May 2017 to April 2018 were reviewed after obtaining approval from our institutional review board (IRB). Patients’ demographics (age, sex, race, BMI) and comorbidities were collected retrospectively from the charts. A total of 18 patients met our inclusion criteria. After propensity-score matching, four patients were included in our final analysis.

Outcomes

Primary Outcomes

**Figure 1:**
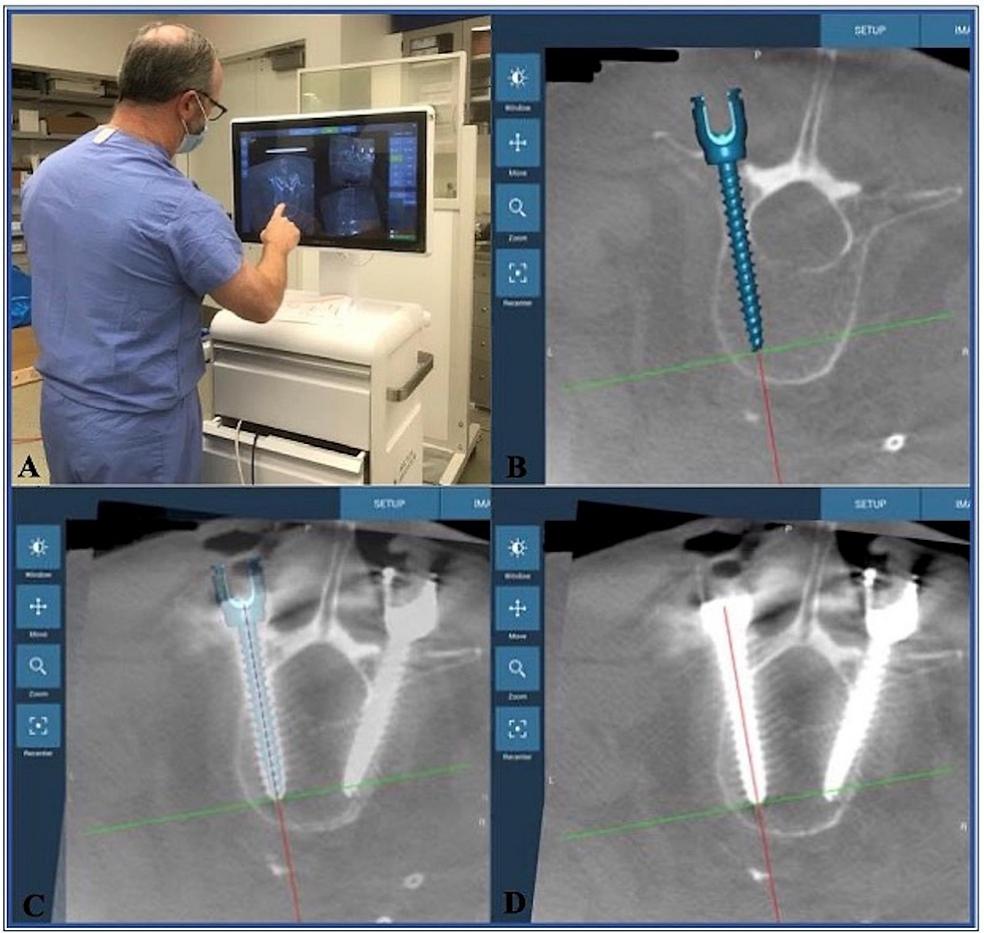
Fusion Robotics™ platform workflow A and B: creating a screw plan; C: checking the accuracy of the screw to the pre-placement plan; D: checking the accuracy of the pedicle screw placement on the post-placement scans

The primary outcome measure of this study was the robotic workflow efficiency (duration) for both groups. The duration of the system set-up, creating a sterile barrier (ready for the scan), scanning and importing images, creating a screw plan (Figures 1A, 1B), plan to in-position for the first screw, screw insertion, and the total duration of the procedure were measured during the lab in the Fusion Robotics^TM^ group and during the surgery in the Mazor-X^TM^ group using a stopwatch.

Secondary Outcomes

The secondary outcomes were the accuracy of the screw to the pre-placement plan ("yes" or "no") (Figure 1C) and the accuracy of the pedicle screw placement on the post-placement scans using either a post-procedure O-arm spin in the Fusion Robotics^TM^ group (Figure 1D) or a postoperative CT scan in the Mazor-X^TM^ group. Pedicle screw placement accuracy was determined using the scale developed by Iampreechakul et al. [10]. If the screw was positioned entirely within the pedicle, it was determined to be grade A; lateral or medial breach of the pedicle wall of <2 mm was deemed grade B; lateral or medial breach of the pedicle wall of 2-4 mm was considered grade C; lateral or medial breach of the pedicle wall of >4 mm was termed grade D [10]. The screw placement accuracy was determined by the senior author.

Statistical analysis

The baseline characteristics (age, sex, race, BMI, comorbidities, and surgical levels) of both groups were compared using Student's t-test and chi-squared tests. The propensity-score matching analysis was performed to compare the intraoperative workflow parameters as well as screw placement accuracy. The propensity score was calculated using the patient baseline characteristics (age, sex, race, BMI, and surgical levels) as independent variables. Furthermore, one-to-one matching was performed to conform to baseline demographics. A case that was performed using Fusion Robotics^TM^ was matched to a case that was performed using the Mazor-X^TM^ Stealth Edition with a nearly similar propensity score. The primary outcomes (workflow durations) were compared using Student’s t-test and the secondary outcomes (screw placement accuracy and accuracy to plan) were compared using the chi-squared test. The statistical analysis was performed using the SPSS Statistics software version 26.0 (IBM, Armonk, NY), and statistical significance was set at a p-value of less than 0.05.

## Results

Demographics

After propensity-score matching, there were four procedures in each group with no significant between-group differences in terms of sex, race, BMI, and surgical levels. However, there were significant differences in terms of age (p=0.01) and comorbidities (p<0.001) between both groups, and this was attributed to the Fusion Robotics^TM^ cases being performed on cadavers of older age and higher comorbidity scores (Table 1).

**Table 1:** Patient demographics *Statistically significant BMI: body mass index; SD: standard deviation

Variables	Fusion Robotics^TM^ group	Mazor-X^TM^ Stealth group	P-value
Number of cases	4	4	
Mean age (years)	87	63 ± 9.3	0.01*
Female gender (%)	50%	75%	0.5
White race (%)	100%	100%	1
BMI (mean ± SD)	25.1 ± 3.2	24.3 ± 2.8	0.7
Charlson Comorbidity Index score (mean ± SD)	6 ± 1	2 ± 0.7	<0.001*
Levels, n (%)			
L2-3	2 (50%)	0	0.1
L3-4	0	2 (50%)	0.1
L4-5	2 (50%)	2 (50%)	1

Primary outcome

**Figure 2:**
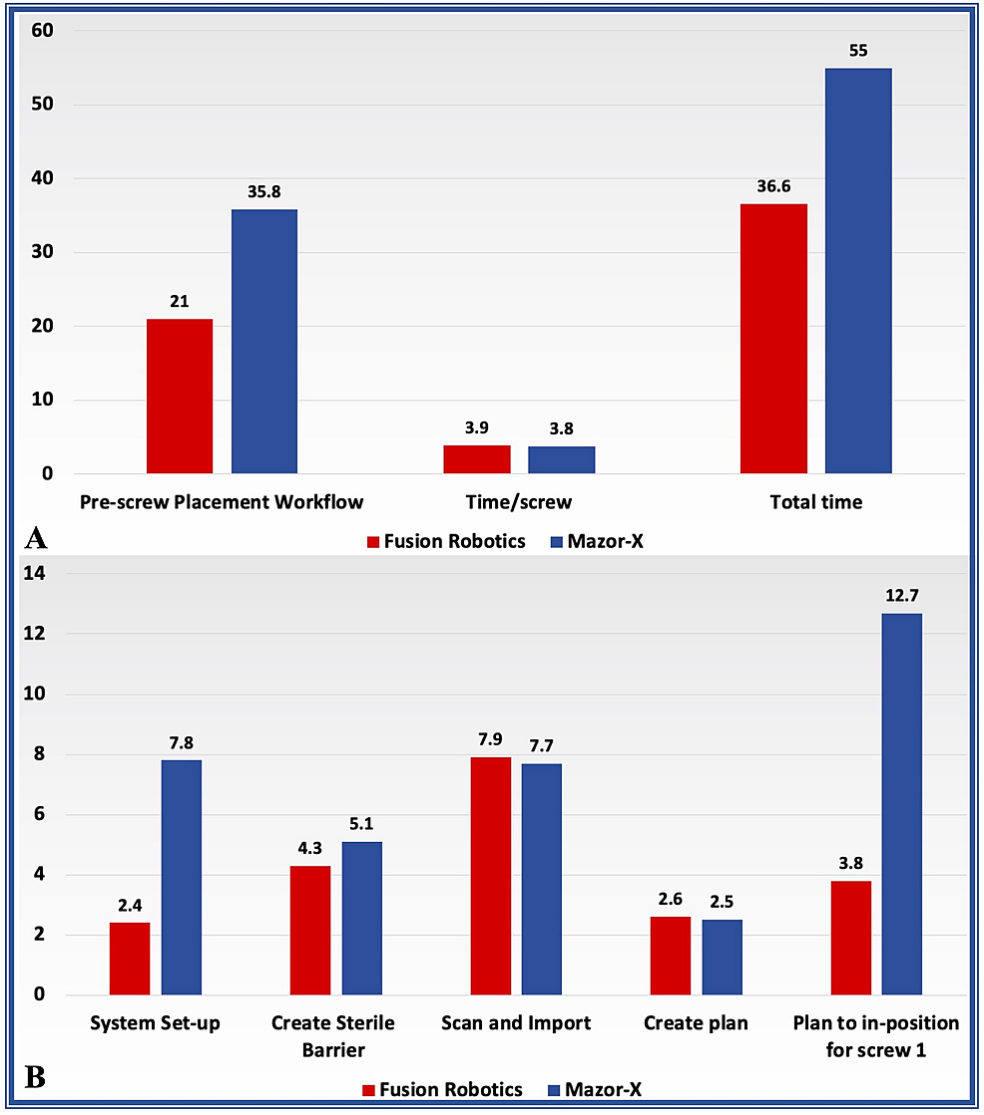
Bar charts comparing the operative workflow efficiency (duration in minutes) in both groups

The surgical workflow efficiency before the placement of the screws was non-significantly longer in the Mazor-X^TM^ group (p=0.1); however, the original system set-up time (p<0.05) and the planning to in-position time (p<0.05) were significantly longer with regard to the Mazor-X^TM^. Furthermore, the total procedure time was significantly longer in the Mazor-X^TM^ group (p<0.05). There was a similar workflow duration in both groups during creating a sterile barrier (p=0.5), scanning and import of images (p=0.9), creating a plan (p=0.8), and screw placement (p=0.9) (Table 2, Figure 2).

**Table 2:** Operative workflow efficiency (duration) in minutes SD: standard deviation; PSIS: posterior superior iliac spine

Procedure	Fusion Robotics^TM ^group, mean ± SD	Mazor-X^TM^ Stealth group, mean ± SD	P-value
System set-up	2.4 ± 0.2	7.8 ± 1.2	<0.05*
Create sterile Barrier "ready for scan"	4.3 ± 1.5	5.1 ± 1.8	0.5
Scan and import	7.9 ± 2.8	7.7 ± 3.1	0.9
Create plan	2.6 ± 0.5	2.5 ± 0.5	0.8
Plan to in-position for screw 1	3.8 ± 0.5	12.7 ± 5	<0.05*
Pre-placement of screw	21 ± 2.1	35.8 ± 14.9	0.1
Screw 1 placed	2.9 ± 0.7	4 ± 1.6	0.3
Screw 2 placed	4.7 ± 1.4	3.8 ± 2	0.5
Screw 3 placed	4 ± 0.5	4.3 ± 2.9	0.9
Screw 4 placed	3.8 ± 0.3	3 ± 0.7	0.1
Time/screw	3.9 ± 1.1	3.8 ± 2	0.9
PSIS pin placement	0.3 ± 0.1	0.3 ± 0.1	1
Total time	36.6 ± 4.4	55 ± 1.9	<0.05*

Secondary outcomes

**Figure 3:**
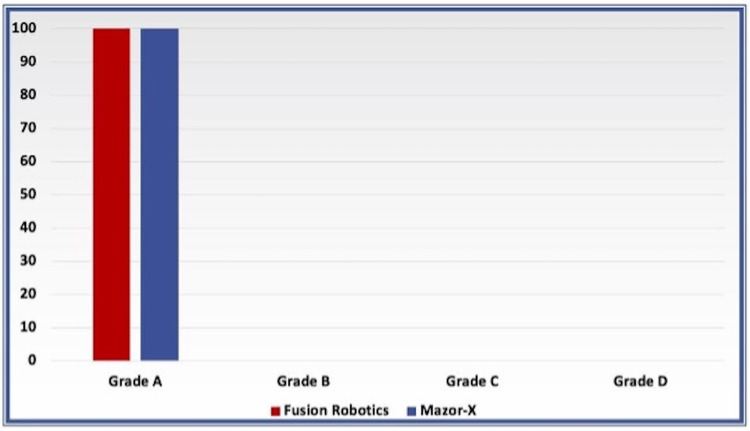
Bar charts comparing the screws placement accuracy in both groups according to Iampreechakul et al. grading

**Figure 4:**
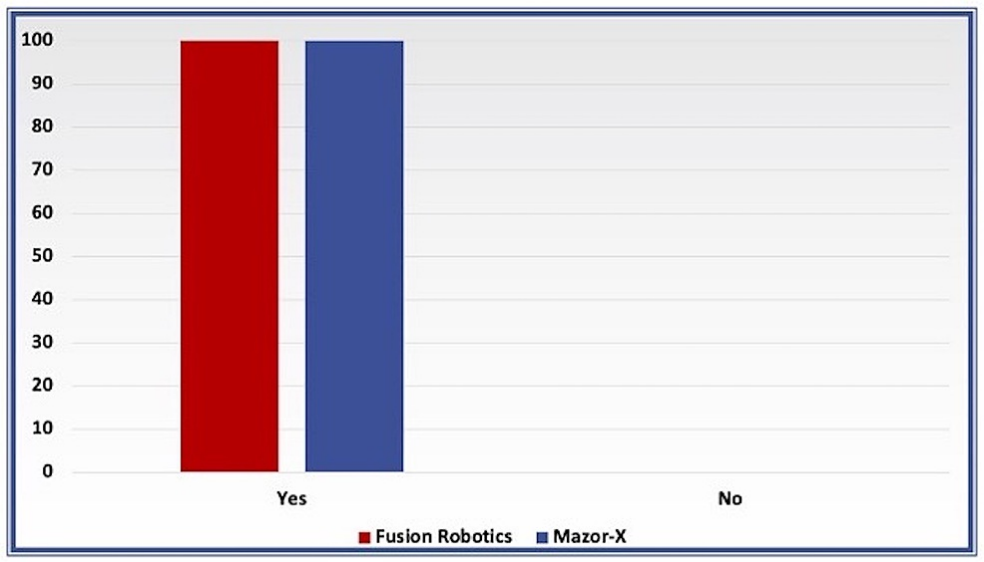
Bar charts comparing the screws’ accuracy to plan in both groups

According to grading based on Iampreechakul et al., all the screws in both groups were determined to be grade A on the post-placement images with no significant difference between groups (Figure 3). Furthermore, all of the screws were accurate compared to the replacement plan with no significant difference (Figure 4).

## Discussion

Fusion is one of the most common surgical procedures for the lumbar spine [11-13]. The incidence of lumbar fusion has increased from 7.5 to 17.8 per 100,000 in 2000 and 2009, respectively [13-14]. To improve and optimize the accuracy of such procedures, real-time intraoperative navigation and robotics technology were developed and have been widely utilized [15]. However, there are few reports comparing the accuracy of the robotic pedicle screw placement to free-hand placement [15-19]. Moreover, there are no prior studies comparing pedicle screw placement workflow efficiency and screw placement accuracy between different spine robotic platforms.

This study aimed to compare the efficiency and accuracy of the Fusion Robotics^TM^ system to those of the Mazor-X^TM^ system for single-level lumbar pedicle screw planning and placement. The primary outcome measure was workflow efficiency. The study results showed that the Fusion Robotics^TM ^platform had a significantly shorter workflow duration in terms of system set-up time, planning to in-position time, and total procedure time. There was no significant difference between the systems in terms of creating a sterile barrier, scanning and importing images, creating a plan, and screw placement. Regarding the secondary study outcomes, there was no significant difference between the platforms in terms of screw placement accuracy and screw accuracy to plan.

Primary outcomes

In this study, where all procedures were performed by the same surgeon, there was a significantly shorter total procedure time for the Fusion Robotics^TM^ group (36.6 ± 4.4 minutes) compared to the Mazor-X^TM ^group (55 ± 1.9 minutes). This significant difference was attributed to longer system set-up time (2.4 ± 0.2 versus 7.8 ± 1.2 minutes) and plan to in-position time for the first screw (3.8 ± 0.5 versus 12.7 ± 5 minutes). The pre-placement of the screw (system set-up) time was non-significantly shorter in the Fusion Robotics^TM ^group (21 ± 2.1 versus 35.8 ± 14.9 minutes). Also, compared to published data regarding the ExcelsiusGPS® robotic platform, the pre-placement screw time (28.1 ± 5.2 versus 21 ± 2.1 minutes) was significantly shorter for the Fusion Robotics^TM ^platform while the total operative time (41.4 ± 8.8 versus 36.6 ± 4.6 minutes) was non significantly shorter [20]. With regard to the screw placement time, there were no significant differences between the Fusion Robotics^TM^, Mazor-X^TM^, and ExcelsiusGPS® robotic platforms [20]. There is no relevant published data regarding workflow efficiency for the ROSA ONE® platform.

Secondary outcomes

Lumbar pedicle screw placement accuracy for both the Fusion Robotics^TM^ and Mazor-X^TM^ groups in this study was 100%. There are many published clinical studies evaluating the screw placement accuracy of the Mazor Robotics platform, with screw accuracy ranging from 92.6 to 99.5% [21-24]. Furthermore, in the present study, all of the screws were accurate compared to the pre-planned trajectories. These results are similar to the results published by Vaccaro et al. using the ExcelsiusGPS® robotic platform, in which 100% of the screws were graded A and B [20]. No significant accuracy differences were seen compared to the ROSA ONE® platform, which showed 97.3% grade A and B screw accuracy in a previously published study [25].

Future robotic platforms

Three robotic control systems are currently used in surgeries: (1) the shared-control system, where the robot and the surgeon perform the surgery simultaneously; (2) telesurgical interaction, where the surgeon remotely controls the surgery done by the robot (such as with the da Vinci system); and (3) the supervisory-controlled system, where the surgeon pre-plans and supervises the operation done autonomously by the robot [7]. All of the previous spine robotic platforms use the shared-control robotic system [7]. One innovation in the Fusion Robotics^TM ^system is that it includes approximately 90% manual surgeon control of the robot. This enhances the range of motion of the robot and makes it more applicable to single-position surgery, whereas other robotic platforms have trouble getting into position. After the manual positioning is completed by the surgeon, the final trajectory is targeted using controls that can be positioned inside or outside the sterile field. This makes the Fusion Robotics^TM ^platform a hybrid system between a shared-control system and a telesurgical interaction system. Other advantages of the Fusion Robotics^TM ^platform include a near-field localization camera attached to the operating room table, which minimizes line-of-sight issues. The small sizes of the robot and camera afford the surgeon more room to move and work throughout the procedure.

Limitations

As with all retrospective studies, there is an information bias risk because this study was partially based on chart review. However, this bias was minimized by propensity score matching of the Mazor-X^TM^ cohort to the four procedures in the Fusion Robotics^TM^ group. Despite the small sample size, there was a significant difference between the groups in terms of the total procedure time, the system set-up time, and the plan to in-position time for the first screw. Furthermore, even though the same surgeon performed all procedures, this was a comparative study between cadaveric surgeries in a lab setting and live cases in an operating room setting.

Another important difference between the groups was that the surgical team had extensive experience using the Mazor-X^TM^ Stealth Edition system involving hundreds of procedures at the time of data collection, whereas the same surgical team had undergone only an approximately 30-minute training session with the Fusion Robotics^TM^ system prior to completing the procedures.

One metric that was collected for the Fusion Robotics^TM^ system but was not available for comparison with the Mazor-X^TM^ system was post-procedure breakdown time. From an overall operating room efficiency and logistics perspective, this metric will be of value to compare robotic platforms in the future. The breakdown time for Fusion Robotics^TM^ System was 2.4 ± 0.3 minutes.

## Conclusions

Based on our findings, the Fusion Robotics^TM^ platform has a significantly shorter procedure workflow duration (better efficiency) with the same accuracy as the most commonly used spine robotic platform (Mazor-X^TM^). In addition, the Fusion Robotics^TM ^system involves manual surgeon control of the robot, thereby enhancing its range of motion compared to other robotic platforms. Thus, it takes a step closer to telesurgical interaction and supervisory-controlled systems. Further clinical studies with a large sample size are required to validate our findings.
